# Is Nursing a Profession?

**Published:** 1919-03-15

**Authors:** 


### IS NURSING A PROFESSION?

Having delivered my soul, as in duty bound, concerning the machinery of the College, I desire to return to a subject which in The Hospital of the 17th ultimo I had only begun to discuss.

Is Nursing a Profession? It became the common law of the nursing world that none save mature women may be permitted to enter the training schools as pupils. It is this law that I take the liberty of challenging. But first of all let it be acknowledged without hesitation that with such hours and such duties as at present fall to the lot of the probationer no lower age limit is conceivable. Neither the hours of the probationer, however, nor her duties are in accord with a professional status, and what I ask of the reader is to consider the subject as though it were to be thrown into the melting-pot, and included in a great scheme of reconstruction. Suppose that an eight-hour day were made to include the probationer's professional lectures and work in the wards; suppose that her academic year were made to correspond with that of a student of medicine—in these circumstances may I ask if the age limit might be reduced to eighteen?

I am for the moment not concerned whether Hospitals can afford to accept probationers on such terms. I am willing to believe that many of them cannot, and that the probationer's sweated labour forms the main pillar of the edifice. But this is quite beside the question. What I want to know is whether with shorter hours and lighter work a nurse can emerge from the schools fully trained at the age of twenty-one or twenty-two.

### Learning the Business of Life.

At present a young woman leaving school either stagnates or follows some other occupation for four or five years, and then begins to learn the business of life. And at the same time it is found possible to make tailors, soldiers, doctors, dentists, barristers, chemists, architects, accountants, and so forth, without any such hiatus. The one reserved service for such unique and extraordinary treatment is Nursing. The Nurse begins late in life to learn, and she retires early. Statistics show that her working life is from fifteen to twenty years, her wages and her economic welfare, during service and after, undergoing corresponding shrinkage.

The status of the entire profession as well as the pay and the outlook are contingent on this very matter. Betterment must begin below in any radical scheme of reform. Any other plan must necessarily be a thing of shreds and patches. Anybody who reads the nursing press must be struck with the manner in which increases of pay to nurses are being doled out. They are mere sops to draw recruits and to keep the establishments staffed. Hence it behoves those of us who are in the habit of referring to "the profession" to look the facts fairly and squarely in the face, and either abandon this title of courtesy or else endeavour to co-ordinate the customs, the pay, the hours and the educational standard with the dignity which is at present purely nominal.

I should like to see this subject discussed without bias or preconceived prejudice. Never mind whether Hospitals can afford to do without their underpaid overworked probationers, and substitute therefore pupils whose object is training rather than toiling. If they cannot, the facts must be presented to the public that these are the people on whose sacrifice the nursing of the nation's sick poor largely depends.

### Educational Status.

I welcome Miss Gladys Leigh's letter. Her first point is, of course, obvious, but it had not been overlooked or forgotten by me. The educational status of a probationer is a very large and important subject, and its consideration was merely postponed. I confess, however, that I am unable to follow Miss Leigh's other arguments, and I think that they require some elaboration.

The subject is one that the College will one day have to tackle. Probably nursing public opinion concerning it is not yet quite sufficiently formed, nor its importance fully realised. It is, indeed, no exaggeration to say that the extent and importance of the economics of nursing is only now becoming generally understood. Two years or more have elapsed since I introduced the subject in the pages of this Journal, and battles royal were then being fought concerning State Registration. If I am any judge of the portents of the weather even the opponents of the College are beginning to realise that Registration was overdone, and that only two things really matter. One of these is the science and art of nursing; the other is the economics of nursing. And these are not separate and independent water-tight compartments. They are interdependent, and nurses will find it necessary to put up a very strong fight in order to raise the standard. There are doctors who profess to prefer badly-educated nurses to women with intellectual attainments. I have my own opinion as to the explanation. But even among nurses there are very few who realise that the fate of the future probationer influences the career of trained women, and it is always difficult to stir up enthusiasm where there is no self-interest. This is a grim truth learnt only in the school of experience. It happens that the fate of the future probationer will in a marked degree affect the career of both sister and matron. And hence I hope that the subject will not be lightly put aside. In the nursing world there are two forces constantly working. One tends to lift the profession to a higher plane—the other to drag it down. It is urgently important that every nurse should clearly discriminate between that which makes for righteousness and that which tends to degrade. This is not always easy. Hence the inestimable value of an organ of public opinion where every argument may be presented for the consideration of the multitude.

IERNE.

